# Supplementary material for: Crawling and Gliding: A Computational Model for Shape-Driven Cell Migration
Source: PLoS Comput Biol. 2015 Oct 21;11(10):e1004280. doi: 10.1371/journal.pcbi.1004280 (PMC4619082; doi:10.1371/journal.pcbi.1004280)
Supplement: S1 Code — (ZIP) [file pcbi.1004280.s012.zip › release/tst/doc/html/sticky_8h_source.html]

Tissue Simulation Toolkit: sticky.h Source File


Tissue Simulation Toolkit
 0.1.4.1


- Main Page
- Namespaces
- Classes
- Files

- File List
- File Members

sticky.h

Go to the documentation of this file.

1 /\*

2

3 Copyright 1996-2006 Roeland Merks

4

5 This file is part of Tissue Simulation Toolkit.

6

7 Tissue Simulation Toolkit is free software; you can redistribute

8 it and/or modify it under the terms of the GNU General Public

9 License as published by the Free Software Foundation; either

10 version 2 of the License, or (at your option) any later version.

11

12 Tissue Simulation Toolkit is distributed in the hope that it will

13 be useful, but WITHOUT ANY WARRANTY; without even the implied

14 warranty of MERCHANTABILITY or FITNESS FOR A PARTICULAR PURPOSE.

15 See the GNU General Public License for more details.

16

17 You should have received a copy of the GNU General Public License

18 along with Tissue Simulation Toolkit; if not, write to the Free

19 Software Foundation, Inc., 51 Franklin St, Fifth Floor, Boston, MA

20 02110-1301 USA

21

22 \*/

23 #define GRIDX 100

24 #define GRIDY 100

25 #define MAXCELLS 1024 /\* beware \*/

26 #define MAXNEIGH 2054

27 #define FALSE 0

28 #define TRUE 1

29 #define OK 1

30 #define TESTCELLS 1

31 #define BOLTZMANN 1024

32

33

34 #define MAXSEED 65531349

35

36 /\* #define SAMPLE POPSIZE/2 \*/

37

38 //#define DEBUG 0

39 #define MAXHIST 4096

40 #define MAXTYPE 256

41 #define WHITE 0

42 #define BLACK 1

43 #define RED 2

44 #define BLUE 3

45 #define GREEN 4

46 #define MEDIUM 0

47 #define EMPTY -1

48 #define DIV 1 /\* DIV 2^(# of divbit) \*/

49 #define REMARK 56

50 #define HASHCOLPRIME 255

51 #define PLOTPERIODFREQUENCY 25

52 #define HOSTDEAD -20

53 #define NULL\_BEAST -10

54 #define OK\_BEAST -5

55

56 /\* the number of external connections per genome

57  of random DNA's \*/

58 /\* "parameters" \*/

59

60 #define PMUT 0.009

61 #define PMUT2 .1

62 #define DMUT 0.0021

63 #define PCO 0.

64 #define NETSIZE 29

65 #define INETSIZE 16

66 #define INP\_PROT 16 /\* number of negative inputs (connectivity) \*/

67 #define POTPROT 16 /\* number of potential proteins \*/

68 //#define EXTCONNECTIONS 0

69 #define COMMUNICATION 2

70 #define ENERGYOFFSET 8

71 #define NHHIST 10

72 #define NH\_TH 0.5

73 #define ENDOFSTATES 1<<(NETSIZE+2)

74 /\* function definitions \*/

75

76 double RANDOM();

77

78

79

80

81

82

83

84

85

86

87

88

RANDOM

double RANDOM()

**Definition:** random.cpp:33


---

Generated on Thu Aug 14 2014 22:04:01 for Tissue Simulation Toolkit by  

 1.8.6
